# The Therapeutic Potential of Pericytes in Bone Tissue Regeneration

**DOI:** 10.3390/biomedicines12010021

**Published:** 2023-12-20

**Authors:** Assel Issabekova, Gulshakhar Kudaibergen, Aliya Sekenova, Aidar Dairov, Madina Sarsenova, Sholpan Mukhlis, Abay Temirzhan, Murat Baidarbekov, Saule Eskendirova, Vyacheslav Ogay

**Affiliations:** 1 Stem Cell Laboratory, National Center for Biotechnology, Astana 010000, Kazakhstan; lsc@biocenter.kz (A.I.); kudaibergen@biocenter.kz (G.K.); sekenova@biocenter.kz (A.S.); dairov@biocenter.kz (A.D.); sarsenova@biocenter.kz (M.S.); muhlis@biocenter.kz (S.M.); eskendirova@biocenter.kz (S.E.); 2 National Scientific Center of Traumatology and Orthopedics Named after Academician N.D. Batpenov, Astana 010000, Kazakhstan; abaytemirzhan@gmail.com (A.T.); baidarbekov_m@nscto.kz (M.B.)

**Keywords:** pericyte, osteogenesis, bone defect, regeneration

## Abstract

Pericytes, as perivascular cells, are present in all vascularized organs and tissues, and they actively interact with endothelial cells in capillaries and microvessels. Their involvement includes functions like blood pressure regulation, tissue regeneration, and scarring. Studies have confirmed that pericytes play a crucial role in bone tissue regeneration through direct osteodifferentiation processes, paracrine actions, and vascularization. Recent preclinical and clinical experiments have shown that combining perivascular cells with osteogenic factors and tissue-engineered scaffolds can be therapeutically effective in restoring bone defects. This approach holds promise for addressing bone-related medical conditions. In this review, we have emphasized the characteristics of pericytes and their involvement in angiogenesis and osteogenesis. Furthermore, we have explored recent advancements in the use of pericytes in preclinical and clinical investigations, indicating their potential as a therapeutic resource in clinical applications.

## 1. Introduction

The regeneration of massive bone defects remains a challenging and not fully resolved problem in traumatology and orthopedics. After bone tumor, surgical removal, or severe trauma, massive bone defects do not recover spontaneously and require a significant amount of bone grafting [1,2]. Currently, for the treatment of massive bone defects, various methods of osteoplasty using autologous and allogeneic osteografts, as well as bone substitutes, are applied [3,4,5]. The application of bone autograft transplantation is considered the gold standard in bone defect treatment. However, this method has some drawbacks and limitations: (1) harvesting of bone from donor sites is a painful procedure and takes a long time to recover; (2) the lack of the required amount of donor bone tissue. In cases where autologous transplantation is difficult, allogeneic transplantation is the most common alternative. The cadaveric bones are used for this application, so additional operations are not required. However, there are some serious disadvantages, which include reduced bone strength during sterilization, graft rejection, and the risk of contracting hepatitis or acquired immunodeficiency syndrome (AIDS). The use of bone substitutes does not always lead to the complete restoration of massive bone defects. At present, great hopes for massive bone defect regeneration are reasonably associated with the use of tissue-engineered biomaterials. For the effective restoration of structural and functional characteristics of the damaged bones, the use of stem cells, growth factors, and biopolymers or scaffolds is suggested. Generally, searching for tissue-engineered methods for the development of an appropriate biomaterial is faced with several issues: the choice of an optimal cell source, the choice of factors stimulating regeneration, and the choice of an optimal biocompatible carrier or matrix. A promising cell source for tissue engineering is adipose tissue (AT), which, in comparison with other tissue sources, contains not only many mesenchymal stem cells but also perivascular stem cells (PSCs) or pericytes [6]. This article highlights studying the pericytes and the therapeutic potential of pericytes combined with a different biological carrier and stimulating factors to accelerate the regeneration of damaged bone tissue.

## 2. Morphological Characteristics of Pericytes

According to classical histology, pericytes (“rouge cells” or “mural cells”) are a population of outgrown cells of connective tissue that surround small blood vessels. The site of the localization of pericytes is the outer wall of the microvessels, blood capillaries, and small venules (postcapillaries) that form a basal membrane with endothelial cells. Pericytes are contractile cells that are similar to the smooth muscle cells (SMCs) of an arteriolar wall. In contrast to SMCs, they are completely embedded in the basal membrane of capillaries and are directly involved in the growth and functional development of a microcirculatory bed. In addition to the structural regulation of the capillary wall, pericytes are involved in the dynamic modulation of microvascular tone and diameter. Researchers identified the unique functions of pericytes that were associated with the regulation of the capillary blood flow, angiogenesis, phagocytosis, and other physiological processes of a microvasculature [7,8,9] (Table 1).

Depending on the localization of the microvascular bed and the degree of differentiation, pericytes exhibit different morphology, ranging from a typical flat, stellate shape (CNS) to a more rounded shape (in the kidney). On average, cells are ~20 µm long and ~0.5 µm thick. In general, pericytes have an elongated shape, a characteristic convex nucleus, and numerous processes. The body of the pericytes is always elongated along the direction of the blood flow. Long primary processes are located along the long axis of the capillary. Short secondary processes encircle the capillaries and form tight junctions with the endotheliocytes. The irregular distribution of the content that includes actins and myosins in the pericytes’ populations that depend on their localization causes notable differences in their contractility [8,9,37,38,39,40,41].

Pericytes regulate the formation of the basal membrane through the secretion of extracellular matrix proteins: collagen IV, laminin, and fibronectin. The interaction between pericytes and endotheliocytes occurs through dense intercellular contact and various signaling pathways. The slotted intercellular junctions (nexus) allow the interaction of pericytes’ cytoplasm with endotheliocytes to exchange substances with the help of connexin proteins by ion transport and diffusion of nutrients and metabolites. Adhesion junctions ensure the transmission of mechanical contractile forces from pericytes to endothelial cells and the involvement of N-cadherin/catenin complexes with fibronectin-based cellular adhesion molecules. On the cytoplasm side, the glycoprotein complexes are bound to the actin filament bundles, which is important for pericyte attachment and movement. The pericyte-endothelial interdigitations provide strong mechanical binding, metabolism, and increase in the area of the intercellular interactions. The intercellular contacts between pericytes and endotheliocytes influence their mitotic activity and gene expression and, consequently, change each other’s phenotype [7,8,39,40,41,42,43]. The density of the pericytes on the surface of the microvessels varies between the different organs. The vascular bed of the nervous system is considered to be the most covered by pericytes (endotheliocyte to pericyte ratio is 1:1–3:1). The much lower endothelial to pericyte ratio (100:1) was described for human skeletal muscle tissue. Pericyte coverage of the vascular surface ranges from 70% to 10%, which is related to the structure of the hematoparenchymal barrier, endotheliocyte proliferation, and vascular diameter. Reduced pericyte density and circumference of the microvessels are responsible for neurodegenerative diseases and uncontrolled angiogenesis [38,39,44,45].

Despite the aforementioned advantages of pericytes, the maintenance and application of these cells hold certain limitations that researchers are actively investigating and attempting to overcome. Among them are heterogeneity, differential behavior, and functional stability. Not all pericytes possess the same regenerative potential as well as cell behavior and functionality that might vary based on the microenvironment and signaling cues they receive. This variability can affect their response and efficiency in tissue regeneration in different conditions. For instance, type-2 pericytes can be recruited and stimulate endothelial cells to form new vessels during tumorigenesis [46].

## 3. Markers of Pericytes

It is known that pericytes are multipotent cells present in all vascularized tissues of the body. The role of the pericytes is not limited to the regulation and maintenance of the microvascular bed; they can also differentiate into phagocytes, chondrocytes, adipocytes, myocytes, and osteoblasts. Numerous studies have established that perivascular pericytes from various human organs and tissues have the differentiation potential and expression profile inherent to mesenchymal stem cells (MSCs) [47,48,49,50]. Both in vivo and in vitro, pericytes express markers typical of MSCs, such as CD44, CD73, CD90, and CD105 [30,51]. Pericytes, which carry the marker antigens of the MSCs, are most effectively detected in blood vessels of the adipose tissue (AT) and dental pulp. It was determined that pericytes can differentiate into immune cells, such as dendritic cells and macrophage-like cells, which play an important role in inflammation and under pathological conditions. Despite similarities in the localization and expression of surface immunomarkers, the ability to differentiate in vitro into osteoblasts, chondrocytes, adipocytes, myocytes, and smooth muscle cells and form foci of ectopic osteogenesis in vivo, the biological equivalence of pericytes with MSCs remains open.

Currently, there is no single molecular marker that can be used to identify the pericytes unambiguously. Difficulties in the identification of them are associated with the fact that all known pericyte markers are used for the phenotyping of other types of human cells. The most known markers of pericytes are smooth muscle alpha-actin (α-SMA), platelet-derived growth factor receptor beta (PDGFR-β), nerve/glial antigen 2 (NG-2), and CD146 (Table 2) [8,9,39,40,45,46,47,48,49,50,52,53].

The α-SMA (alpha-smooth muscle actin) marker is used to identify pericytes because they are identified as microvascular analogs of smooth muscle cells of the blood vessels. The α-SMA marker is the highly conserved contractile protein of smooth muscle cells that involves vasoconstriction and vasodilation of the vessels. The expression of α-SMA by capillary pericytes is significantly lower than in arterial smooth muscle cells, which was related to the rate of the regulation of blood pressure for vascular wall contraction. A mid-capillary pericytes with thin and spindle-shaped cytoplasmic processes do not express α-SMA, whereas pre- and postcapillary pericytes with thick and stellate processes are rich in a-SMA expression [9,37,38,39,45,46,47,48].

NG2 (neural/glial antigen 2, chondroitin sulfate proteoglycan 4—*CSPG4*) is an integral membrane proteoglycan that participates in transmembrane signal transduction, cell adhesion, intercellular communication, migration, and proliferation. This marker is actively expressed by pericytes of arterioles and capillaries but is absent in pericytes of the venules. NG2 maintains the vascular network homeostasis, and its absence in venous vessels contributes to the regulation of arterial/venous anastomoses. However, NG2 expression is also characteristic of actively proliferating cells: activated macrophages, chondroblasts, osteoblasts, keratinocytes, fibroblasts, and some tumor cell types (gliomas and melanomas) [8,9,37,38,39,43,47,48,49,50].

For accurate phenotyping of NG2-expressing pericytes, an expression assay for the PDGFRβ marker is used. The PDGFRb (Platelet-derived growth factor receptor beta)—PDGFRb is a tyrosine kinase receptor that enables the differentiation of pericytic precursor cells. It is the most detectable marker of microvascular pericytes that are functionally involved in the recruitment of pericytes for normal blood vessel development and stabilization during angiogenesis. Dysregulation of the PDGFRβ kinase activity is responsible for the development of cardiovascular diseases [37,38,39,42,44,45].

CD146 (melanoma cell adhesion molecule, MCAM) Mel-CAM, MUC18, S-endo1 is a transmembrane glycoprotein with a molecular weight of approximately 110 kDa. It functions as a Ca^2+^-independent cell adhesion molecule and is connected with intercellular interactions. It was reported that CD146 can promote pericyte recruitment with endotheliocytes through the direct regulation of the PDGF-B/PDGFRβ signaling pathway. Thus, this marker is presented as a universal marker of the brain pericytes, bone marrow, myocardium, and skeletal muscle. The authors have established an important role for CD146-positive pericytes in the control of the blood–brain barrier [9,38,39,44,45,46,47,48,49,50,52,53].

The aforementioned markers are notably expressed in pericytes at different stages. The NG2 and α-SMA markers are predominantly expressed by mature pericytes, whereas PDGFR-β is expressed by pericytic progenitor cells. Expression of the NG2 and α-SMA correlates with the type of vessels they surrounded. The pericytes enveloping capillaries are NG^2+^/α-SMA−, pericytes of the venules are NG2−/α-SMA+, pericytes of the arterioles are NG^2+^/α-SMA+, whereas PDGFR- and CD146 are ubiquitously expressed in all types of pericytes. Such evidence is related to stages of pericyte differentiation, specific tissue structure, pathological condition, vascular hierarchy, and stages of development [9,37,38,42,48,49,50,52,53].

The other markers that are used to identify pericytes are specific within the organ or tissue. For example, CD13 (aminopeptidase N), a type II membrane metalloprotease, is a marker of cerebral pericytes and is associated with the blood–brain barrier. ALP (alkaline phosphatase), an enzyme involved in metabolism, is actively expressed by skeletal pericytes. The regulator of G-protein-5 signaling (RGS5) is a protein that is expressed by activated pericytes during vascular remodeling and active tumor development. CD34 is a transmembrane phosphoglycoprotein that participates in cell adhesion as well as in the regulation of differentiation and proliferation. The CD34 marker is presented as a marker of the adventitial pericytes subpopulation. The expression of a nestin (cytoskeleton intermediate filament protein) marker by pericytes was found to be of two types, NG^2+^/Nestin− and NG^2+^/Nestin+, which differ in their angiogenic ability. The NG^2+^/Nestin+-positive pericytes that promote angiogenesis are targeted for anti-angiogenic therapy [7,8,9,42,43,49,50]. 

Unlike endothelial cells, pericytes do not express the next markers CD31, CD34, CD144, vW (von Willebrand factor).

## 4. A Role of Pericytes in Angiogenesis

The localization of pericytes in the vascular space indicates that the endothelium is an important component of the perivascular niche capable of regulating the functional activity of pericytes. Pericytes play a key role in angiogenesis and are involved in the regulation of blood flow, vessel formation, remodeling, and stabilization. Determining the mechanisms that regulate these processes is crucial for understanding the pathogenesis of vascular complications.

Many soluble factors and signaling pathways are known to be fundamental for mutual intercellular interaction and communication between pericytes and endothelial cells (EC) at the molecular level. Pericytes are involved in the formation and sprouting of new blood vessels by signaling molecules such as PDGFR-b, transforming growth factor beta (TGF-b), vascular endothelial growth factor (VEGF), angioprotein 1 (Ang-1) and sphingosine-1-phosphate (S1P) [7,8,9,37,38,39,40,42,44,45,46,47].

Under hypoxic conditions, pericytes release VEGF-A to activate and transform endothelial cells (tip cells) and stimulate their migration during vascular sprouting. For pericytes’ adhesion (recruitment) on new vessels, PDGF-B secreted by sprouting endothelial cells binds to the pericyte-specific receptor PDGFRβ. The importance of PDGF/PDGFRβ signaling for capillary stabilization has been demonstrated in endothelio-specific PDGF-B knockout mice, resulting in endothelial hyperplasia, aberrant vasculature, and microaneurysms. Stabilization of the newly formed blood vessels is further regulated by the Ang-1/Tie-2 signaling pathway, in which Ang-1, a ligand produced by pericytes, binds to the endothelium-specific receptor Tie-2. Ang-1 is a natural inhibitor of vascular permeability. The Ang-1/Tie-2 binding promotes the association of pericytes and endothelium to form a tight vascular barrier.

The activation of the angiopoietin-1 antagonist, angiopoietin 2 (Ang-2), which binds to the Ang-1 receptor (Tie-2), contributes to vascular instability. The blocking of Ang-1-Tie-2 signaling leads to weakened contact between endothelial cells and pericytes, which increases their permeability and leads to vascular regression. Thus, the balance of the Angpt1/Angpt2 ratio determines vascular homeostasis and is essential for the regulation of new vessel formation and maturation [9,37,38,39,40,42,43,45,47,60,61,62,63].

The connection of pericytes with endothelial cells activates transforming growth factor beta (TGFβ), which inhibits endothelial cells (EC) proliferation and migration but stimulates the differentiation of pericytes and hence promotes blood vessel maturation. The importance of TGFβ is supported by studies in which the absence of TGFβ in experimental mice led to abnormal vascular network development, which was characterized by a lack of pericyte coverage, vascular instability, and rupture [7,8,9,37,38,40,47,53,61].

In addition, the interaction between ECs and pericytes is regulated by the S1P signaling molecule, a sphingolipid, which is involved in cell communication. The S1P maintains the vascular endothelial barrier by promoting the secretion of extracellular matrix components, enhancing the interaction of the connective proteins, N-cadherin and VE-cadherin, and inhibiting vascular destabilization factors such as Angpt2 and VEGF-A [9,38,47,61].

Thus, pericytes are important regulators of the angiogenesis process within definite signaling pathways that form the normal vascular bed.

**Figure 1:**
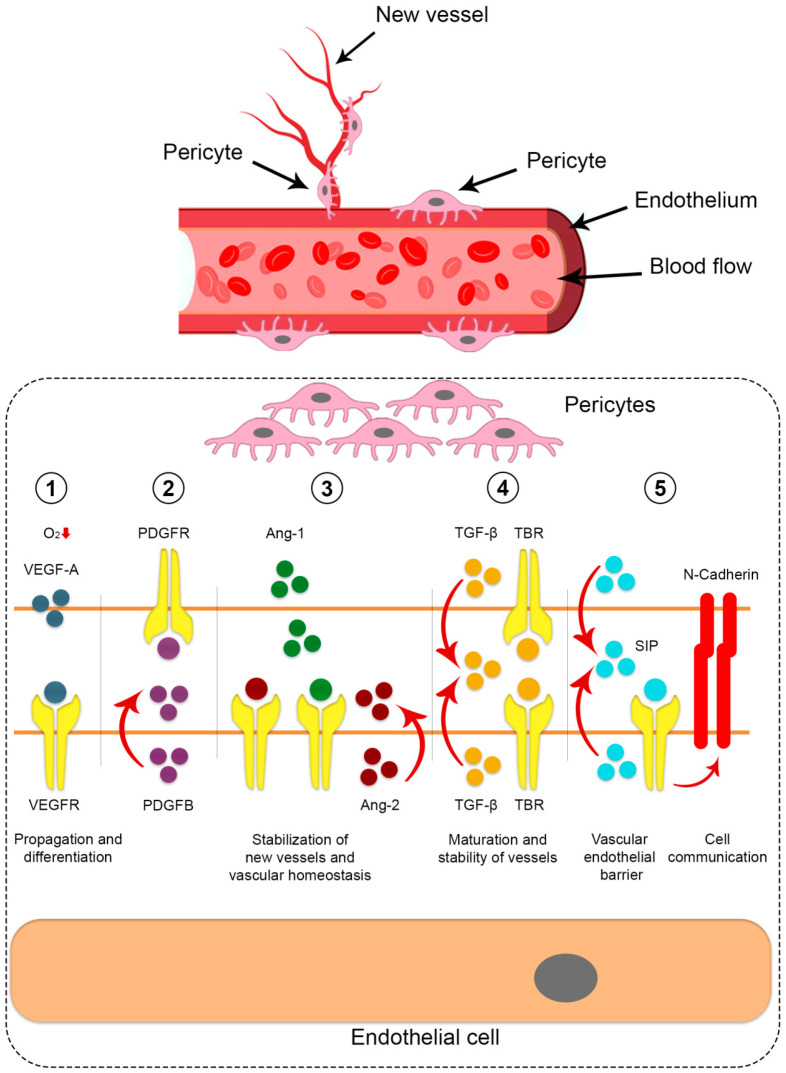
Cell signaling pathways between pericytes and endothelial cells, which are involved in the process of angiogenesis. (**1**) VEGF, secreted from pericytes, is implicated in propagation and differentiation processes; (**2**) Proliferation and migration of pericytes occur by PDGF-B, obtained from endothelial cells; (**3**) Ang-2/Tie-2 signaling ensures stabilization of new vessels and vascular homeostasis; (**4**) TGF-β that produced by both pericytes and endothelial cells, mediate the processes of maturation and stability of vessels; (**5**) S1P from endothelial cells maintain the vascular endothelial barrier. N-cadherin preserves cell communication between pericytes and endothelial cells.

This review of the current scientific data shows that researchers in the field of regenerative medicine have considered the pericytes as a promising therapeutic target. However, there are still many challenges, requiring the careful study of the identification of pericytes, the mechanism of pericyte differentiation, the determination of phenotypic differences between pericytes in angiogenesis and mature vessels, and the detailed determination of signaling pathways. Also, it is necessary to understand the functions of pericytes in the microcirculatory stream and the possibilities of their use for regenerative medicine (Figure 1).

## 5. Pericytes in Osteogenesis

Different cell types such as MSCs from various sources, osteoblasts and osteoclasts, embryonic stem cells (ESCs), induced pluripotent stem cells (iPSCs), chondrocytes, adipose-derived stem cells (ADSCs), and periosteum-derived stem cells were investigated for bone regeneration by different researchers. From these studies, it is clear that bone marrow (BM) MSCs remain the most widely used progenitor cells since the discovery of this multipotent cell population [63]. However, the aforementioned cells have several limitations. For example, obtaining MSCs from older individuals or those with certain health conditions might impact their regenerative properties. Direct use of osteoblasts and osteoclast cells may be limited due to challenges in obtaining and expanding them in culture. Osteoblasts might not maintain their functionality over prolonged culture periods, affecting their efficacy in bone repair. Regarding the ESCs and iPSCs, the ethical concerns and the risk of teratoma formation, as well as potential genetic abnormalities induced during reprogramming in iPSCs, are major limitations associated with cells. Obtaining other cell types, such as ADSCs, might involve invasive procedures while harvesting. Moreover, their differentiation potential can vary between donors, impacting their regenerative capabilities. Additionally, chondrocytes, even primarily involved in cartilage formation, possess limited ability to form bone and face challenges in maintaining their phenotype during expansion in culture, restricting their utility in bone tissue engineering [64]. In this regard, considering the drawbacks of various cell types for bone tissue regeneration, pericytes can be a promising source for this goal.

The potential of pericytes in osteogenesis was studied in an experimental animal model of muscle pockets, calvaria, spine fusion, and nonunion fracture [18]. As reported, the significant potential for bone regeneration was found for CD146+ positive pericytes/progenitors, and the optimal cell source was the subcutaneous adipose tissue (AT). The most meaningful effect in ossification for ectopic bone formation showed the combination of human AT-derived pericytes with osteoinductive demineralized bone matrix (DBM) with increased VEGF [18]. Therefore, the cell therapy by pericytes for spinal fusion in combination with the DBM scaffold resulted in notable osteodifferentiation and complete bone regeneration due to the paracrine mechanism. For the treatment of nonunion fractures, the CD146+ AT pericytes showed prominent bone healing; however, the complete regeneration depended on how attractive the microenvironment of the injured site was for pericyte shifting [18]. The potential mechanism of the effect of pericytes for bone healing was formulated: close ossification of transplanted cells, activation of the shifting and proliferation processes in osteoprogenitor cells, and paracrine and immunomodulatory effect on osteogenic and endothelial cells [25]. The authors reported that pericytes from human AT showed significant regeneration of the damaged bone in rats’ model of osteoporosis in the next combinations: within the bone chips and sodium hyaluronate-based scaffold, joined with the NELL-1 protein or with the gel foam-alginate 3D scaffold [65]. Also, for calvarial injuries, pericytes within the scaffold from poly(lactic-co-glycolic-acid) (PLGA) scaffold, from alginate gel, from collagen sponge or hydroxyapatite-based polymeric scaffold demonstrated the effect of regeneration [65] (Table 3). Thus, reported studies confirm the high osteogenic potential of pericytes in the acceleration of bone defects’ healing due to paracrine factors, immunomodulation, and vascularization.

Thus, pericytes play a crucial role in bone tissue regeneration. These multifunctional cells are located around blood vessels and have been identified as a promising cell type for supporting tissue repair and regeneration. Understanding the specific mechanisms by which pericytes contribute to bone regeneration is a fascinating area of study that holds promise for future therapeutic interventions in orthopedics and regenerative medicine.

## 6. Recent Preclinical and Clinical Application of Pericytes

A long-practiced approach widely used in clinics to facilitate fracture healing is demineralized bone matrix (DBM) implantation [71]. Among DMB implantation methods, iliac crest autografts and fibula allografts are widely used in clinical practice to repair various fractures. However, in 10–25% of cases, these approaches are not effective in repairing delayed union and nonunion in elderly patients [72]. Moreover, these procedures are known to have several adverse effects, not only during the initial surgery but also years later, due to issues like scars, numbness, and pain that can impact quality of life [73]. As an alternative strategy, cellular therapy with pericytes can be proposed [27]. Bone is an organ with an extensive vascular system that ensures efficient blood flow and nutrient delivery. Therefore, the use of mural cells as pericytes for bone tissue repair has long been posited, as these cells are essential in bone development and regeneration. Experimental evidence has established that pericytes are the endogenous precursors of mesenchymal stem cells [51]. Pericytes can be isolated from AT, which, compared to other tissue sources, also contains many mesenchymal stem cells and can relatively easily promote the acquirement of substantial quantities of the cells [6,74].

Recent animal studies reported that smooth muscle actin (SMA) after a fracture also participates in the formation of bone cells from endogenous mural cells [75]. SMA is a non-specific marker that is additionally expressed on smooth muscle and fibroblasts/myofibroblasts. Therefore, it cannot directly confirm this phenomenon. Studies have focused on identifying populations of pericytes that possess the highest potential for osteoblast formation. One of the study results demonstrates that CD146+ pericytes obtained from skeletal tissue have the greatest potential for the repair and formation of bone both in vitro and in vivo compared with ones obtained from adipose or dermal tissue [76]. However, subsequent clinical studies confirmed the ability of AT pericytes to regenerate bones [63]. For successful isolation of pericytes from many tissues, the CD146+CD34−CD31−CD45−CD56− immunophenotype can be used [77]. Among soft-tissue pericytes, cells expressing CXCR4 have a high osteoblastic potential, which is classified as non-adipocytic progenitor cells [76]. NG2, CD146, and PDGFRβ-positive pericytes in lineage-tracing experiments using NG2-Cre or tamoxifen-induced NG2-CreER mouse strain revealed that they can differentiate into osteogenic cells in mice [26]. Pericytes labeled with tdTomato expression form callus in fractured femurs and have a positive expression of Runx2 and type I collagen corresponding to bone tissue in a bone fracture model [26]. In addition, for successful bone regeneration, the primary cartilaginous callus must be replaced by a hard bone callus. This process involves molecules of the Wnt family, which regulate the differentiation of pluripotent MSCs into the osteoblastic lineage. Subsequently, at later stages of development, the formation of osteoblasts is regulated. As chondrocytes in the fracture callosum proliferate, they become hypertrophic, and the extracellular matrix becomes calcified. This cascade is driven by macrophage colony-stimulating factor (M-CSF), receptor activator of nuclear factor kappa B ligand (RANKL), osteoprotegerin (OPG), and TNF-a. The focus of homogeneous nucleation and formation of apatite crystals are deposits of calcium and phosphate, which appear after the formation of calcium granules and their transportation into the extracellular matrix, where they precipitate with phosphate and form primary mineral deposits. By day 14, peak callus formation occurs, which is determined by histomorphometry of mineralized tissue, as well as by measuring extracellular matrix markers such as type I procollagen, osteocalcin, alkaline phosphatase, and osteonectin. As hard callus formation progresses and calcified cartilage is replaced by woven bone, the callus becomes harder and mechanically rigid [78].

Thus, pericytes isolated from AT demonstrate significant bone-forming potential in all autologous and xenogenic experiments in vivo. Additionally, recent studies have demonstrated that pericytes derived from human white AT with positive expression of alkaline phosphatase and bone matrix have bone-formation potential in an intramuscular mouse model [53].

Another issue in cellular-based therapy is the choice of a method for delivering cells into a defect area. Cell delivery can be done in a single or multiple dose or using scaffolds, which are then implanted into a bone defect.

**Figure 2:**
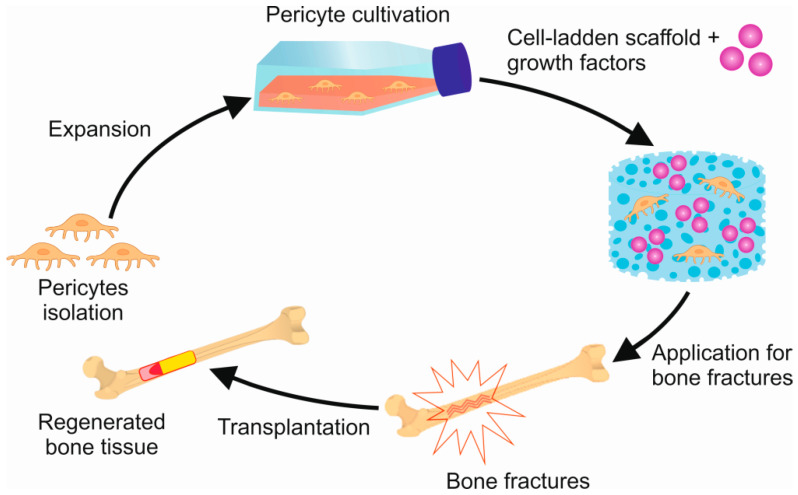
Pericytes and Growth Factors Based Scaffold for Bone Regeneration.

Polymers find extensive application in tissue engineering as scaffolds for cell cultures. These particular polymers are referred to as “cell-laden scaffolds”. The application of such polymers is based on the ability to create a three-dimensional tissue structure, followed by the incorporation of cells and the introduction of growth factors, cytokines, or chemokines into the scaffold [79]. The scaffolds should not only serve as a matrix for cells but should promote cell proliferation and differentiation. Additionally, these polymers must possess specific properties, such as non-toxicity, biodegradability, biocompatibility, and non-immunogenicity [80]. Demineralized bone matrix grafts, synthetic and natural polymers, bioactive ceramics, cross-linked hydrophilic hydrogels, metals, and composite scaffolds are often used as cell-laden scaffolds to retain cells and control the release of bioactive factors (Figure 2) [75].

PSCs have been the subject of investigation in the context of scaffolds in various preclinical models to explore their osteogenic potential using DBM [74,81,82,83], collagen sponge [66], hydrogel [84,85], bone matrix [68,69,70], or polymer coated hydroxyapatite [6].

Several animal models in ectopic bone formation confirmed the potential of implanted human pericytes for osteogenic differentiation in vivo. The results of the study demonstrated the formation of inconspicuous bone after AT pericyte implantation with a carrier of a collagen sponge in a muscle [66]. In another work, AT pericytes were implanted using an osteoinductive DBM carrier, which resulted in strong bone formation in muscle pocket osteogenesis [66]. Other researchers have reported similar findings, that CD146+ AT-derived cells did not show significant bone formation when implanted in a subcutaneous model. This is in contrast to CD146+ precursor cells from the bone marrow or periosteum, which exhibited strong bone tissue formation [86]. Comparisons of AT-derived pericytes, adventitial cells, and an unsorted/uncultured stromal population (termed stromal vascular fraction, SVF) showed that adventitial cells have similar bone-forming potential and SVF is not effective enough [53,66]. SVF or pericytes in combination with adventitial cells (PSC) from the same patient on a hydroxyapatite-coated polymeric scaffold in a mouse calvarial defect model revealed that PSC led to a significant increase in bone regeneration [66]. Additionally, AT-derived human PSC using a DBM carrier in a rat spinal fusion model resulted in paracrine-mediated bone formation [69].

Several studies by James et al. confirmed the effectiveness of pericyte application in bone disease treatment [25,42,53,74,87]. In particular, the research group proved that human AT pericytes incorporated in the PLGA scaffold accelerated the healing of critical-size skull defects in mice within 2 weeks [6].

Another interesting research conducted by Zhang and colleagues suggested a novel pre-vascularization strategy using a tri-culture of human induced pluripotent stem-cell-derived mesenchymal stem cells (hiPS-MSCs), human umbilical vein endothelial cells (HUVECs) and pericytes on calcium phosphate cement (CPC). CPC scaffolds were designed to possess 100–300 μm macropores. Six groups were tested in in vitro and in vivo research. According to the obtained results, bi-culture and tri-culture groups exhibited the formation of vessel-like structures in vitro, and in vivo study showed that the tri-cultured group exhibited a significantly larger quantity of new bone than bi-culture, mono-culture, and CPC control [88,89]. Thus, as shown by a multitude of preclinical studies, pericytes may show promising results in clinical applications in the future [25].

Concerning modern clinical practice, bone-healing therapies are based on the local delivery of cells to the fracture site in combination with growth factors and scaffolds (Table 4).

Potential osteoinductive growth and differentiation factors in combination with pericytes are used for optimal bone formation. Among the growth factors, the recombinant bone morphogenetic protein-2 (BMP-2) is known to be used in fusion facilitation [90]. Another factor, recombinant human platelet growth factor (rhPDGF), is used to accelerate the process of bone defect filling [91]. Additionally, several studies demonstrated the application of (BMP-2) [66], NELL-1 [66,67,86,92], the short isoform of NELL-1 [93] or WNT16 [94], which are used as osteoinductive factors for bone tissue restoration. Although all these factors are osteoinductive, the mechanism of action is different. For example, NELL-1 promotes both bone and cartilage formation, while WNT16, or the short isoform of NELL-1, has combined mitogenic/pro-osteogenic effects [91].

Recombinant BMP-2 is clinically available, and its combination with PSC induces a synergistic effect in bone formation [25,95]. Mumcuoglu and colleagues developed an injectable hydrogel based on collagen microspheres and alginate to deliver BMP-2 into bone defects [96]. Other researchers, taking into account the heparin-binding ability of BMPs, mixed heparin with carriers such as chitosan, poly-L-lactic acid, and DBM [97]. Similar studies were conducted by Yang et al., who developed an injectable fibrin hydrogel for long-term delivery of BMP-2 through covalent conjugation of heparin to fibrinogen and showed its effectiveness in bone defect regeneration [98].

Jing Bai and colleagues have developed an organ-specific microfluidic platform recapitulating the in vivo angiogenic microenvironment by co-culturing mouse primary brain endothelial cells with brain pericytes in a 3D collagen scaffold [99]. Morrison K. et al. fabricated a prevascularized scaffold containing a hierarchical vascular network. Human pericytes and fibroblasts were encapsulated into the polydimethylsiloxane (PDMS) mold containing the 3D Pluronic F127 microfiber and macrofiber network. After 28 days of cultivation, investigators revealed that neoangiogenic sprouts formed in the collagen protodermis and pericytes self-assembled around both fabricated vessels and neoangiogenic sprouts [100]. Other researchers designed a PDMS device with a reservoir for a 3D fibrinogen gel with pericytes. It allows the interaction of endothelial cells (ECs) with pericytes and the extracellular matrix (ECM) in full bio-matrix-encased 3D vessel structures (neovessels) [101].

Currently, clinical studies have been conducted for the restoration of bone defects using adipose-derived mesenchymal stem cells (Ad-MSC) and a preparation based on autologous microfragmented adipose tissue (Table 5), which have shown promising results. The use of an enriched fraction of pericytes may potentially yield better outcomes than merely halting the progression of the disease, as demonstrated in the clinical use of Ad-MSC and autologous microfragmented adipose tissue.

Thus, studies on pericyte application with growth factors in cell-laden scaffolds have shown that the use of scaffolds or any other matrices for pericytes has great potential in restoring damaged bone tissue. The study of other properties of pericytes, such as pro-osteogenic, pro-vasculogenic, and immunoregulatory functions, which have not yet been studied, may give further impetus to preclinical and clinical studies.

## 7. Conclusions

Human pericytes are a unique population of perivascular cells with mesenchymal stem-cell properties present in all vascularized tissues. Pericytes have a high ability for multilineage differentiation, especially in the osteogenic direction. Given the enormous role of pericytes in the stabilization and formation of microvessels and angiogenesis, pericytes are of particular interest in the regeneration and engineering of vascularized bone tissue.

Efficient biomaterials and appropriate manufacturing techniques play a critical role in the development of injectable hydrogels that function as scaffolds for bone tissue engineering. Over the past 10 years, a variety of injectable hydrogels have been developed. These biomaterials include gelatin, alginate, collagen, chitosan, poly-L-lactic acid, hyaluronic acid, fibrin, heparin, and polyethylene glycol [114].

The application of various in vivo bone-formation models based on injectable hydrogels and polymer scaffolds with pericytes, which include ectopic muscle pocket osteogenesis and calvarial replenishment in mice, spinal fusion in rats and dogs, and fracture healing in rats, confirmed the therapeutic efficacy of perivascular cells for accelerated osteoregeneration.

Thus, although scientists have made progress in a cellular-based approach for bone regeneration, there is still a necessity to advance the development of improved injectable hydrogel using stem cells and/or osteoinductive factors for efficient regeneration of damaged bone tissue.

## Figures and Tables

**Table 1:** Physiological and Pathological Functions of Pericytes.

Function	Comments/Reference
*Physiological*	
Blood–brain barrier (BBB)	BBB is a specialized vascular structure that restricts the passage of most molecules from the systemic circulation into the central nervous system (CNS). It is crucial for proper neuronal function and is maintained by various cell types, collectively known as the neurovascular unit [10]. Pericytes, found along capillary walls, play a vital role in BBB maintenance, immune cell regulation, and brain blood flow control within the CNS [11]. They are part of the neurovascular unit, which manages interactions between neurons and cerebral blood vessels to meet the brain’s energy needs [12]. Loss of pericyte coverage can lead to BBB dysfunction and the accumulation of neurotoxic molecules, impacting white matter lesions [13]. The interaction between endothelial cells and brain pericytes can induce BBB characteristics during embryogenesis and is used in in vitro BBB models [14].
Vascular permeability	Increased pericyte coverage on tumor vasculature reduces vessel permeability, limiting the entry of pro-inflammatory and pro-tumorigenic cells [15]. In the context of hemorrhagic shock, pericytes act as protective cells situated on the basolateral side of the endothelium, playing a crucial role in maintaining vascular barrier function in pulmonary and peripheral vessels [15,16].
Vaso- constriction	Pericytes play a key role in regulating blood flow in microvessels. They exhibit contractility through proteins like α-SMA, desmin, vimentin, and C-GMP, which affect actin filament bundles near endothelial cells. Pericytes respond to vasoconstrictors (e.g., angiotensin-II, serotonin) and vasodilators (e.g., nitric oxide, cholinergic agonists, adenosine) by changing the collagen lattice’s surface area in vitro [9]. In rat cortex slices, ischemia causes vasoconstriction near pericytes, followed by pericyte death, suggesting their involvement in blood flow regulation and a potential role in reperfusion after ischemia [17].
Angiogenesis	Pericytes, found in microvessels like capillaries and venules, play a vital role in maintaining structural integrity, regulating blood flow, promoting angiogenesis, stabilizing vasculature, and controlling permeability. In the central nervous system and retina, they form barriers protecting cells from harmful blood factors [18]. Pericytes are crucial for angiogenesis, influencing vessel stability by interacting with sprouting endothelial cells. They can also adjust capillary diameters, similar to smooth muscle cells, regulating microvessel blood flow [19].
Osteogenic	Pericytes can cause osteochondral differentiation and paracrine-induced osteo- and vasculogenesis and have immunomodulatory effects [20]. As a cellular source of osteogenic cells, endogenous pericytes differentiate into osteoblasts and osteocytes and aid in the healing of bone fractures [21,22]. Microvascular pericytes can produce bone, cartilage, and fibrous tissue, in particular, pericytes positive for NG2, CD146, and PDGFRβ [20,23]. Moreover, pericytes can induce bone mineralization and fusion, as well as increase the size of fracture callus [24].
Impact on immune function	Pericytes facilitate immune cell migration by releasing molecules, recruiting various immune cells, and promoting M2-like macrophages. They regulate the immune system in the central nervous system. Reduced CD4+ T-cells lead to decreased pericyte coverage, and retinal pericytes inhibit CD4+ T-cell activation [19].
Stem cell	Pericytes maintain blood vessel integrity, prevent issues like vessel dilation and hemorrhaging, and exhibit stem-cell-like qualities [25]. In the brain, vascular pericytes are crucial for the blood–brain barrier and demonstrate stem-cell capabilities, particularly after brain damage from conditions like ischemia and hypoxia [26,27]. After ischemia, they can transform into various cell types, including neurons, microglia, and vascular cells, and assist in clearing damaged areas by becoming glial cells [28].
*Pathological*	
Fibrosis	Pericytes contribute to lung diseases like pulmonary arterial hypertension (PAH) and allergic asthma by transforming into scar-forming myofibroblasts, which lead to tissue fibrosis through collagen deposition and matrix remodeling [29].
Neuro- degeneration	Pericytes in the blood–brain barrier degenerate in Alzheimer’s disease (AD), which is associated with neurovascular dysfunction, Aβ elevation, tau pathology, and neuronal loss [30]. Pericyte loss in neurological disorders increases blood–brain barrier permeability and may lead to vascular dementia [31].
Cancer	Pericytes in the tumor microenvironment have diverse roles, including forming the pre-metastatic niche, promoting cancer cell growth and drug resistance, and influencing M2 macrophage polarization [32]. In carcinogenesis, disrupted interaction between PCs and endothelial cells leads to dysfunctional tumor vasculature [33]. Recent studies employing advanced technologies confirm pericytes’ communication with cancer cells [34].
Diabetic Retinopathy	Pericyte loss is an early hallmark of diabetes-related microvascular diseases, including retinopathy and nephropathy. Pericytes actively contribute to vascular dysfunction by secreting pro-angiogenic factors, initiating neovascularization, thickening the basement membrane, and causing vasoconstriction [35].
HSCs maintenance	CD34−/CD45−/CD146+ perivascular cells support stemness in human HSCs ex vivo, but unfractionated MSCs and CD146− cells induce differentiation, jeopardizing HSCs’ ability to engraft [36]. CD34−/CD45−/CD146+ cells were effective in sustaining hematopoiesis from both BM and nonhematopoietic adipose tissue acting via cell-to-cell contact mediated by Notch ligands [36]. This highlights a ubiquitous ability of pericytes not restricted to the BM.

**Table 2:** Characteristics of Pericytes from Different Sources.

Species, Tissue	Markers	Reference
Human retina	NG2+, CD146+, CD271+ and CD140B+	[54]
Human fetal skeletal muscle, human fetal pancreas, human placenta, human umbilical cord, and other tissues	CD146+, CD34−, CD45− and CD56−	[51]
Human skeletal muscle	CD146+, CD34−, CD45−, CD56−	[55]
Human adipose	CD146+, CD31−, CD34−, CD45−	[56]
Human canine adipose	CD146+, CD34−, CD45−	[57]
Human bone marrow	CD146+CD34−CD45−	[36]
Mouse skeletal muscle	NG2/DsRed+, Nestin/GFP+/−	[46]
Mouse hepatic tissue	CD146+, CD34−, CD45−, CD56−	[58]
Mouse brain	CD13+, CD31−, CD41−, CD45−	[59]

**Table 3:** Preclinical Studies for Bone-Healing Therapy Using Adipose Pericytes.

Model	Type of Cells and Tissue Origin	Animal Type	Dosage	Method of Investigation	Therapeutic Outcome	Reference
Intramuscular ectopic bone model	Adipose human perivascular stem cells (hPSCs) and adventitial cells derived from the same patient	Severe combined immunodeficient (SCID) mice	2.5 × 10^5^ cells, sponge size 2.0 × 1.0 × 0.5 cm	Micro-CT imaging (bone mineral density and bone volume)	Both perivascular populations had a similar baseline osteogenic potential	[53]
Intramuscular ectopic bone model	Adipose hPSCs, SVF from the same patient	SCID mice	2.5 × 10^5^ cells, sponge size 2.0 × 1.0 × 0.5 cm	High-resolution radiographic/3-Dimensional Micro-CT imaging	Larger particles of bone formation, increase in vascularity of the implant site, osteocalcin, and bone sialoprotein expression	[66]
3 mm non-healing calvarial defect centered in the parietal bone	Adipose hPSCs or SVF from the same patient	SCID mice		Radiographic imaging and histological examination	Significant bone defect healing over time	[6]
Intramuscular ectopic bone model	Adipose hPSCs	SCID mice	The demineralized bone matrix (DBX), with NELL-1 (3 μg/μL), hPSC (2.5 × 10^5^ cells), or hPSC+NELL-1	Micro-CT imaging, histological examination, immunohistochemical staining over 4 weeks	The additive effect of hPSC+NELL-1 on bone formation and vasculogenesis	[67]
Intramuscular ectopic bone model in the hindlimb	Adipose hPSCs or SVF from the same patient	SCID mice	DBX with SVF or hPSC	Histological examination, immunohistochemical staining	Significantly greater neutrophilic and macrophage infiltrates within and around SVF in comparison to PSC-laden implants, robust immunomodulatory effect	[68]
Tibial model	Adipose hPSCs, bone marrow MSC	Wistar rats	5 × 10^6^ cells were percutaneously injected into the fracture gap	Radiographic, micro-CT imaging, and immunohistochemical staining	At eight weeks, 80% of animals in the cell treatment groups showed evidence of bone healing compared to only 14% of those in the control group	[29]
Posterolateral lumbar spinal fusion model	Adipose hPSCs	Athymic rats	DBX, 0.15 × 10^6^ hPSCs, 0.50 × 10^6^ hPSCs, 1.5 × 10^6^ hPSCs	Micro-CT imaging, immunohistochemical staining	Regulate bone formation via direct and paracrine mechanisms	[69]
Osteoporotic spinal fusion mode	Adipose hPSCs or NELL-1, BMP-2	Athymic rats	0.25 × 10^6^ cells per milliliter of hPSCs or 33.3 μg/mL of NELL-1, 0.75 × 10^6^ cells per milliliter of hPSCs or 66.6 μg/mL of NELL-1	Micro-CT imaging, histological examination, immunohistochemical staining	The hPSC combined with NELL-1 synergistically enhances spinal fusion in osteoporotic rats	[70]

**Table 4:** FDA Approved Therapies for Bone Healing.

Systemic	Local
Bisphosphonates	INFUSE (rhBMP-2)
Recombinant parathyroid hormone	Regranex (rhPDGF-BB)
RANKL inhibitors	rhBMP-7 *
SOST inhibitors (pending)	Healos (GDF-5) (pending)
	Demineralized bone matrix
	Fibula allograft
	Iliac crest autograft

* FDA humanitarian device exemption in 2003, failed to pass FDA approval in 2009 (https://www.fda.gov/ accessed on 12 July 2023).

**Table 5:** Features of Clinical Studies of Stem Cells from Fat Tissue.

Dose (Cell) (×10^6^), Donor, Type of Study	Age, Kellgren-Lawrence Grade, Sample (M:F), Follow-Up	Outcome, Measures, Effect	Study (Year)
1 × 10, 2 × 10, 5 × 10, Autologous adipose tissue-MSC (Ad-MSC) In this single-site, randomized, double-blind, dose-ranging, phase I study	18–70 2–3 grade 22 (3:19) samples 48 weeks	WOMAC, VAS, WORMS, MRI, others MRI assessments showed slight improvements in the low-dose group	[102]
50 × 2x, Autologous Ad-MSC Randomized double-blind phase IIb clinical trial	18–70 1–3 grade 52 (6:46) samples 12 months	WOMAC, VAS, MRI, others 50% improvement in WOMAC 70% improvement rate in the Re-Join^®^ group after 12 months	[103]
100×, autologous Ad-MSC Double-blinded, randomized controlled phase IIb clinical trial	18–75 2–4 grade 24 (12:12) samples 6 months	KOOS, WOMAC, VAS, MRI, others Improvement of WOMAC score at 6 months No significant change in cartilage defect in the MSC group	[104]
100, 100 × 2x at baseline and 6 months, autologous Ad-MSC Randomized controlled trial (RCT)	>18 2–3 grade 30 (16:14) samples 12 months	KOOS, NPRS, WOMAC, others Significant pain and functional improvement in both treatment groups	[105]
3.9 (Progenza 3.9 M, *n* = 8) or placebo (*n* = 2) and 6.9 (Progenza 6.7 M, *n* = 8) or placebo (*n* = 2), allogeneic Ad-MSC Double-blinded RCT	40–65 1–3 grade 20 (16:4) samples followed up for 12 months	Significant improvement in VAS and WOMAC pain subscale scores No decrease in average lateral tibial cartilage volume	[106]
6 mL, intra-articular injection, autologous adipose tissue (AT) or hyaluronic acid Prospective, single-center, parallel-group RCT	45–75 2–3 grade 54 (male and/or female, 27:27) 6 week and 6 months	WOMAC, WOMAC-A, PROMIS, force plate analysis, others	[107]
10 mL intra-articular injection, autologous, microfragmented AT or isotonic saline (placebo) Blinded RCT	18–70 2–3 grade 120 patients Primary outcome after 6 months, secondary outcomes after 3, 6, 12 and 24 months	KOOS4, Tegner activity score, work status, others	[108]
Intra-articular knee injection of autologous microfragmented lipoaspirate (MLA)	30–81 12 (6:6)	Stromal vascular fraction isolation, flow cytometry	[109]
4–15 mL intra- articular injection, autologous microfragmented AT containing Ad-MSCs Prospective, non-randomized, interventional, single-center, open label clinical trial	40–85 3–4 grade 17 patients 3, 6, 12, and 24 months after treatment	dGEMRIC, VAS A single intra-articular injection of autologous microfragmented AT improves glycosaminoglycans (GAG) content on a significant scale	[110]
Intra-articular injection, autologous MLA Prospective, non-randomized study	40–85 3–4 grade 20 patients 12 months after treatment	VAS, WOMAC, KOOS KOOS score improved from 46 to 176% when compared with the baseline WOMAC decreased from 40 to 45% VAS rating decreased from 54% to 82%	[111]
Intra-articular injection, autologous microfragmented AT	54–78 3–4 grade 17 patients 6 weeks, 6 and 12 months after treatment	NPRS, 100-point KSS with FXN and LEAS KSS score improved from 74 to 82 FXN score improved from 65 to 76 LEAS score improved from 36 to 47	[112]
Intra-articular injection, autologous and microfragmented AT Retrospective study	54–78 2–4 grade 30 patients 12 months after treatment	KOOS, IKDC, Tegner Lysholm knee, VAS IKDC-subjective and total KOOS improved by 20 points VAS-point and Tegner Lysholm knee improved by 24 and 31 points, respectively	[113]

## Data Availability

The data presented in this study are available on request from the corresponding author.
